# Kangaroo mother care for clinically unstable neonates weighing ≤2000 g: Is it feasible at a hospital in Uganda?

**DOI:** 10.7189/jogh.08.010701

**Published:** 2018-06

**Authors:** Melissa C Morgan, Harriet Nambuya, Peter Waiswa, Cally Tann, Diana Elbourne, Janet Seeley, Elizabeth Allen, Joy E Lawn

**Affiliations:** 1 Department of Paediatrics, University of California San Francisco, San Francisco, California, USA; 2 Maternal, Adolescent, Reproductive, and Child Health Centre, London School of Hygiene and Tropical Medicine, London, UK; 3 Department of Medical Statistics, London School of Hygiene and Tropical Medicine, London, UK; 4 Department of Paediatrics, Jinja Regional Referral Hospital, Jinja, Uganda; 5 Maternal, Newborn and Child Health Centre of Excellence, School of Public Health, College of Health Sciences, Makerere University, Kampala, Uganda; 6 Department of Public Health Sciences, Karolinska Institutet, Stockholm, Sweden; 7 Department of Neonatal Medicine, Institute for Women’s Health, University College London, London, UK; 8 Department of Infectious Disease Epidemiology, London School of Hygiene and Tropical Medicine, London, UK; 9 Medical Research Council/Uganda Virus Research Institute, Uganda Research Unit on AIDS, Entebbe, Uganda; 10 Department of Global Health and Development, London School of Hygiene and Tropical Medicine, London, UK

## Abstract

**Background:**

Kangaroo mother care (KMC) for *stable* neonates ≤2000 g (g) is associated with decreased mortality, sepsis, hypothermia, and length of stay compared to conventional care. The World Health Organization states that KMC “should be initiated… as soon as newborns are *clinically stable*” [12]. However, the majority of deaths occur in *unstable* neonates. We aimed to determine the proportion of admitted neonates meeting proposed instability criteria, assess the feasibility of providing KMC to unstable neonates, and evaluate the acceptability of this intervention to parents and providers at Jinja Regional Referral Hospital in Uganda.

**Methods:**

This was a mixed-methods study. We recorded data including birthweight, chronological age, and treatments administered from medical charts, and calculated the percentage of clinically unstable neonates, defined as the need for ≥2 medical therapies in the first 48 hours of admission. We enrolled a sample of neonates meeting pre-defined instability criteria. Mothers were counselled to provide KMC as close to continuously as possible. We calculated the median duration of KMC per episode and per day. To explore acceptability, we conducted semi-structured interviews with parents and newborn unit care providers, and analysed data using the thematic content approach.

**Findings:**

We included 254 neonates in the audit, 10 neonates in the feasibility sub-study, and 20 participants in the acceptability sub-study. Instability criteria were easily implementable, identifying 89% of neonates as unstable in the audit. The median duration of individual KMC episodes ranged from 115 to 134 minutes. The median daily duration ranged from 4.5 to 9.7 hours. Seventy-five percent of interviewees felt KMC could be used in neonates concurrently receiving other medical therapies. Barriers included lack of resources (beds/space, monitoring devices), privacy issues, inadequate education, and difficulties motivating mothers to devote time to KMC. Recommendations included staff/peer counselling, resources, family support, and community outreach.

**Conclusions:**

There remains a need for an evidence-based approach to consistently define stability criteria for KMC to improve care. We found that KMC for unstable neonates weighing ≤2000g was feasible and acceptable at Jinja Hospital in Uganda. Randomised controlled trials are needed to demonstrate the effect of KMC on survival among unstable neonates in low-resource settings.

Each year, 15 million babies are born preterm (<37 completed weeks gestation) and 1 million deaths occur as a direct result of complications of preterm birth [1-3]. Sub-Saharan Africa and South Asia account for three-quarters of the 2.7 million neonatal deaths that occur annually, and preterm birth is the leading cause of these deaths [4]. Progress in preventing preterm birth has been limited, but major reductions in mortality could be achieved by improving care in low-resource settings [1,3,5,6]. In such settings, 50% of neonates born at 32 to 34 weeks gestation, a time when nearly all should survive, die because newborn special care is not available [3,7]. Kangaroo mother care (KMC) consists of early, continuous skin-to-skin contact (SSC), usually with the infant’s mother; improved breastfeeding; and supportive care for neonates [8,9]. The most recent Cochrane review and a meta-analysis demonstrated that KMC among *stable* neonates ≤2000g is associated with decreased mortality (relative risk (RR) 0.60-0.64) [10,11], sepsis (RR 0.35-0.53) [10,11], hypothermia (RR 0.22-0.28) [10,11], and length of stay (LOS, mean difference -1.61 days) [10] compared to conventional care. WHO guidelines recommend KMC for “routine care of newborns weighing ≤2000 grams (g)… initiated as soon as newborns are clinically *stable*” [12].

However, most neonatal deaths occur in clinically *unstable* neonates within 48 hours of birth in settings without intensive care [3,7]. The only randomised controlled trial (RCT) of KMC in unstable neonates with mortality outcomes was conducted in Ethiopia (123 neonates ≤2000g) and reported major mortality impact (RR 0.57) [13]. Notably, this trial excluded >50% of eligible neonates, did not utilise allocation concealment, and had an apparent imbalance in gestational age and birthweight between groups (favouring KMC) at baseline [13,14]. Among 17 RCTs (14 enrolled only clinically stable neonates) comparing KMC with conventional care in low birthweight (LBW, <2500g) neonates aged <15 days, there was significant variability in how clinical stability was defined. Six defined this based on therapies [15-20], five on ‘hemodynamic stability’ [13,21-24], and three on specific vital sign parameters [25-27], while three provided no definition at all [28-30]. Hence codifying stability criteria for KMC is critical. A recent WHO guideline for care of preterm neonates highlighted the evidence gaps regarding KMC effect on mortality in *unstable* neonates. These included absence of criteria to identify which neonates are *stable enough* to safely receive KMC; the optimal time for initiation; and the duration required to reduce mortality [12].

Compared to other regions of the world, sub-Saharan Africa has experienced slow progress towards reducing neonatal mortality, particularly mortality due to preterm birth [2]. This is likely due to higher preterm prevalence and lower access to care [3,7], and shortages in health workers [31,32]. Further, many interventions are introduced to low-resource settings without adequate evidence of their effectiveness in such settings [3,31]. Incubators, the standard mode of thermal support for small and preterm neonates, are often unavailable or fail to function due to resource-related difficulties such as inconsistent electricity supply or access to replacement parts. Further, they require regular disinfection; however, this is often not done in resource-constrained settings [33]. Other potential issues include risk of cross-infection from other neonates when incubators are shared, and cost [34-36].

In Uganda alone, an estimated 45 000 newborn deaths occur annually, a quarter of which are due to complications of prematurity [37]. As a result, “national attention for maternal and child health has been clear and authorised from the highest levels” [38]. In 2006, the Ugandan government established a Newborn Steering Committee, which advised immediate action to increase the scale-up of KMC in facilities [38].

The purpose of this study was to explore the feasibility and acceptability of KMC for clinically unstable neonates weighing ≤2000g at Jinja Regional Referral Hospital in Uganda. Specifically, we aimed to:

1. determine the proportion of admitted neonates meeting proposed clinical instability criteria,
2. assess the feasibility of monitoring and providing interventions to unstable neonates ≤2000g in the KMC position, and
3. evaluate the acceptability of KMC for unstable neonates ≤2000g to parents and healthcare providers.

## METHODS

### Study setting

This study was conducted at Jinja Regional Referral Hospital, a facility in southeastern Uganda with a catchment area of 4 million. Jinja Hospital has ~ 6500 deliveries annually [39], and preterm birth is common. Sick and preterm/LBW neonates are cared for in the newborn unit, which admits approximately 1200 neonates annually. This unit is distinct from the postpartum ward, where healthy newborns receive care. KMC is employed for neonates deemed *stable* by newborn unit staff. Mothers participate in the care of their babies by providing KMC, feeding (breastfeeding and nasogastric/cup feeds), and checking temperature. The standard mode of thermal support for unstable neonates is incubators, frequently with several neonates sharing an incubator.

### Study design

This was a mixed-methods study consisting of three parts: an admissions audit, a feasibility sub-study, and an acceptability sub-study.

#### Admissions audit

Clinical instability was defined *a priori* as need for ≥2 medical therapies (oxygen, continuous positive airway pressure (CPAP), intravenous (IV) fluids, IV antibiotics, aminophylline, phenobarbital, or phototherapy) during the first 48 hours after birth. The audit aimed to determine the percentage of admitted neonates meeting the proposed criteria. A study coordinator and research assistant were trained on audit objectives and procedures. The target sample size was 250 neonates, based on the suggestion of Sackett et al [40] that 10 charts per variable are needed to obtain accurate and clinically useful results in a retrospective audit. Records were randomly selected across the 12-month audit period (June 2015 to May 2016). We retrospectively recorded data from the medical charts of neonates who were born at Jinja Hospital and admitted to the newborn unit within 48 hours of birth. The research assistant recorded birthweight, date and time of birth and admission, and treatments administered during the first 48 hours of admission. We excluded charts that were not satisfactorily complete, defined as including both birthweight and medical therapies administered, and those with birthweight >2000g from the analysis. To ensure data quality, the study coordinator randomly selected 10% of neonatal charts included in the audit, and double-entered data from those charts into data collection forms. We calculated the frequency and percentage of medical therapies received during the first 48 hours with 95% confidence intervals (CI), and the percentage of neonates meeting the proposed therapy-based instability criteria in that period.

#### Feasibility sub-study

Using instability criteria defined in the audit, we sought to demonstrate the feasibility of monitoring and providing clinical care and interventions (such as oxygen, IV fluids) to unstable neonates in the KMC position. The research team (paediatrician, nurse, study coordinator) and newborn unit nurses had training on the study objectives and procedures. Between July and December 2016, we enrolled a purposive sample of 10 neonates meeting the following eligibility criteria: 1) live-born at Jinja Hospital; 2) birthweight ≥700g and ≤2000g; 3) singleton; 4) chronological age <48 hours; 5) mother able and willing to participate in KMC; 6) infant unstable (defined as in the audit) at the time of enrolment, and followed them until discharge (up to a maximum of 14 days). We selected a small sample size due to the exploratory nature of the intervention and the inherent clinical risks in this vulnerable population. A KMC overview handout was provided and the study nurse counselled mothers during the consent process and throughout the study to provide KMC as close to continuously as possible with a goal of 18 hours per day. For purposes of this study, we defined a KMC episode as beginning when skin-to-skin contact (SSC) commenced and ending when SSC stopped. For interruptions due to mothers carrying out activities like bathing, a family member was encouraged to take over KMC. If none were available, the baby was placed in an incubator until the mother returned. Neonates were managed according to unit guidelines. The study paediatrician oversaw clinical decisions about enrolled neonates. All neonates received continuous monitoring of oxygen saturation (SpO_2_) and heart rate (HR) using Masimo Rad-8 pulse oximeters. Monitoring was commenced at the time of KMC initiation and was continued throughout enrolment. To avoid interruption of KMC, a Bilisoft® phototherapy blanket (GE Healthcare, Chicago, IL, USA) was utilised to treat jaundice. The study nurse and unit nurses completed a data collection form, which recorded date and time of birth and admission, gestational age, birthweight, admission and discharge diagnoses, treatments administered, timing and duration of KMC episodes, date of breastfeeding initiation, LOS, and discharge disposition. Gestational age was calculated by last menstrual period (LMP). When LMP was unavailable, the study nurse conducted a Ballard examination [41]. We calculated the median duration of KMC per episode (minutes) and per day (hours) with interquartile ranges (IQR), and the mean number of concurrent medical interventions delivered per day with standard deviation (SD). Statistical analyses for the admissions audit and feasibility study were carried out using Stata 13 (StataCorp LP, College Station, TX, USA).

#### Acceptability sub-study

We assessed the acceptability of KMC for unstable neonates ≤2000g to parents and providers at Jinja Hospital. The sub-study utilised qualitative research methodology through semi-structured interviews with 20 key stakeholders. In qualitative research, the correct sample size is one that satisfactorily answers the research question [42]. Purposive sampling was utilised to select 10 parents of singleton neonates that were alive and hospitalised in the newborn unit at the time of the interview. All 10 newborn unit providers were included. Interviews took place between May and July 2016. Two interview guides were developed in English - one for parents and one for providers. The guide for parents was translated from English to Lusoga, the local dialect, and translated back to English to ensure accuracy and equivalence [43]. Interviews with parents were conducted in the language with which they were more comfortable. Interviews with providers were conducted in English. The interview guide employed open-ended questions about a broad range of potential factors while allowing the interviewer to ask additional questions on emerging themes. One female interviewer conducted the interviews in a private setting in the language of the participant’s choice. Data validity was supported by the fact that the interviewer was a local woman who spoke the local language. The interviewer was familiar with techniques of qualitative research and had previous experience interviewing parents in a hospital setting. The protocol and interview guide were discussed in detail with the interviewer to ensure she understood the study objectives. Pilot interviews were conducted to identify and revise unclear interview questions and provide additional training on areas of weakness. Consent was requested to audiotape the interview. Interviews were held in a private room to provide confidentiality.

### Thematic analysis

Interview data were transcribed and, where necessary, translated to English. The interviewer performed transcription with the assistance of an experienced Ugandan transcriptionist based at Makerere University. To improve quality control, a sample of early transcripts was compared to the audiotaped interviews to detect transcription errors and correct them. Data were analysed using the thematic content approach [44,45], which consisted of four steps: 1) familiarisation; 2) identifying codes and themes; 3) developing a coding scheme and applying it to the data; and 4) organising codes and themes. The principal investigator (PI) and interviewer read all transcripts and developed the preliminary coding scheme together. Two interviews were double coded by the PI and the interviewer, and any discrepancies were discussed and resolved to develop the final coding framework. The PI coded the remaining interviews. New themes that emerged outside the coding framework were also included in the analysis [46,47].

### Ethical issues

All participants in the feasibility study received standard care available in the newborn unit at Jinja Hospital, including oxygen, CPAP, IV fluids, antibiotics, phototherapy, nasogastric feeds, and anti-convulsant and other medications as clinically indicated and available. Mechanical ventilation was not available. Following a full explanation about the study by the trained nurse (feasibility study) or interviewer (acceptability study) who spoke the local language, written informed consent was obtained from a parent/guardian (feasibility study) or the participant (acceptability study). Ethics Committee approvals were obtained from Makerere University, the Uganda National Council for Science and Technology, the London School of Hygiene and Tropical Medicine (LSHTM), and the University of California, San Francisco (UCSF).

## RESULTS

### 1) Admissions audit

**Figure 1:**
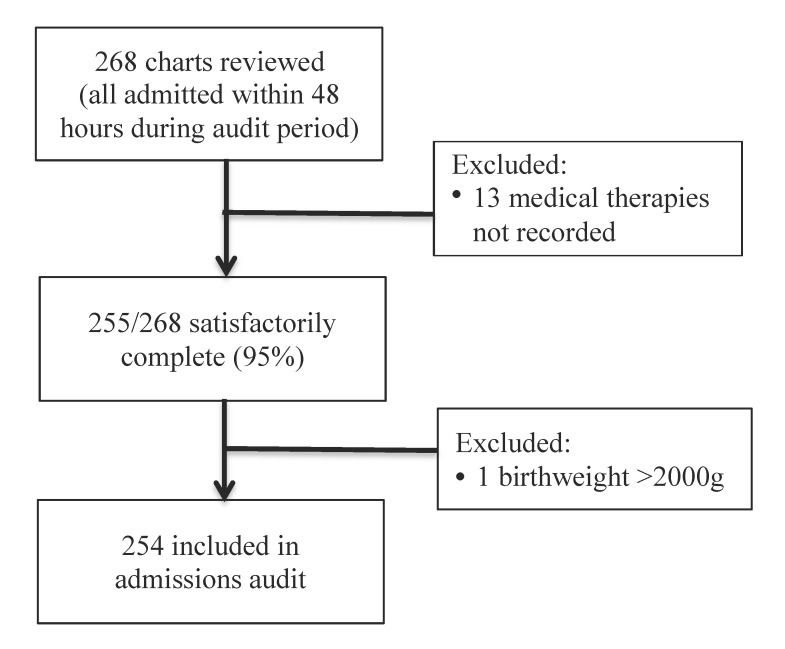
Flowchart showing inclusions and exclusions for admissions audit.

A total of 268 charts were reviewed, among which 255 were satisfactorily complete and 254 met birthweight criteria (**Figure 1**).

Among the 254 included neonates, the mean birthweight was 1477g (SD 318g, range 500-2000g) and 139 (60%) were female. **Table 1** shows the frequency and percentage of all neonates, very low birthweight (VLBW, 1001-1500g) neonates, and extremely low birthweight (ELBW, 500-1000g) neonates with 95% CI by the number of medical therapies received during the first 48 hours after birth. Among all enrolled neonates, 226 (89%, 95% CI: 84.5-92.5) met the proposed instability criteria (≥2 medical therapies). Similarly, 99 (90%, 95% CI: 82.8-94.9) of VLBW neonates and 26 (90%, 95% CI: 72.6-97.8) of ELBW neonates met criteria for instability (**Table 1**).

**Table 1:** Number of medical therapies received among neonates in admissions audit (N = 254)

Number of medical therapies received*	Frequency (%) of all neonates, N = 254	95% CI	Frequency (%) of VLBW neonates, N = 110	95% CI	Frequency (%) of ELBW neonates, n = 29	95% CI
0 to 1	29 (11.4)	7.8-16.0	11 (10.0)	5.1-17.2	3 (10.3)	2.2-27.4
2	73 (28.7)	23.3-34.7	32 (29.1)	20.8-38.5	4 (13.8)	3.9-31.7
3	105 (41.3)	35.2-47.7	47 (42.7)	33.3-52.5	19 (65.5)	45.7-82.1
4 to 5	47 (18.5)	13.9-23.8	20 (18.2)	11.5-26.7	3 (10.3)	2.2-27.4

CI – confidence interval, VLBW – very low birth weight, ELBW – extremely low birth weight

*Oxygen, CPAP, IV fluids, IV antibiotics, aminophylline, phenobarbital, phototherapy.

The instability criteria were easily implementable, leading to clear and timely identification of a substantial proportion of admitted neonates as unstable.

### 2) Feasibility sub-study

**Table 2** shows the participant characteristics. Among the 10 neonates enrolled, median birthweight was 1310g, median gestational age was 28 weeks, median age at enrolment was 25.3 hours, and median LOS was 10 days. Eight neonates were discharged to home, all of whom were breastfeeding (3 with supplemental expressed breastmilk) at the time of discharge. Two neonates died during the study period; both were extremely premature (26-27 weeks) and extremely low birthweight (700-750g).

**Table 2:** Feasibility study participant characteristics (N = 10)

Participant characteristics	Median (IQR)	Range
Birthweight (g)	1310 (820-1600)	700-1800
Gestational age (weeks)	28 (26-31)	26-35
Age at enrolment (hours)	25.3 (4.8-43.9)	1.7-47.1
LOS (days)	10 (9-14)	3-40
	Number	Percent
Female	7	70
Discharged	8	80
Died	2	20

IQR – interquartile range, LOS – length of stay

**Figure 2:**
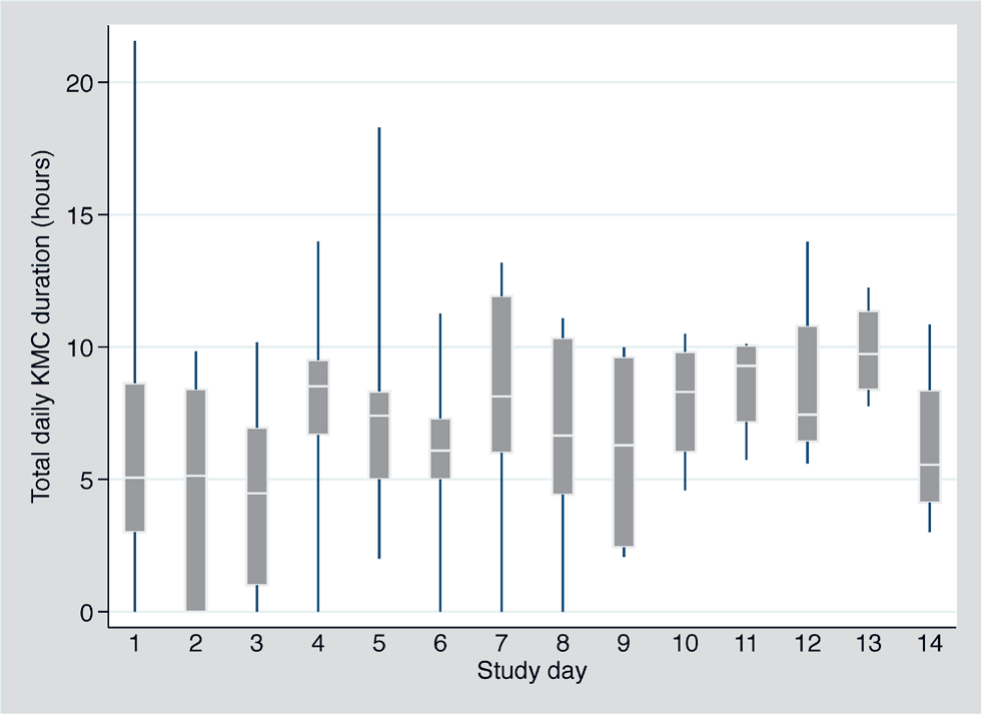
Daily kangaroo mother care (KMC) duration (median, IQR, 5th-95th percentiles) by study day (N = 10).

**Figure 3:**
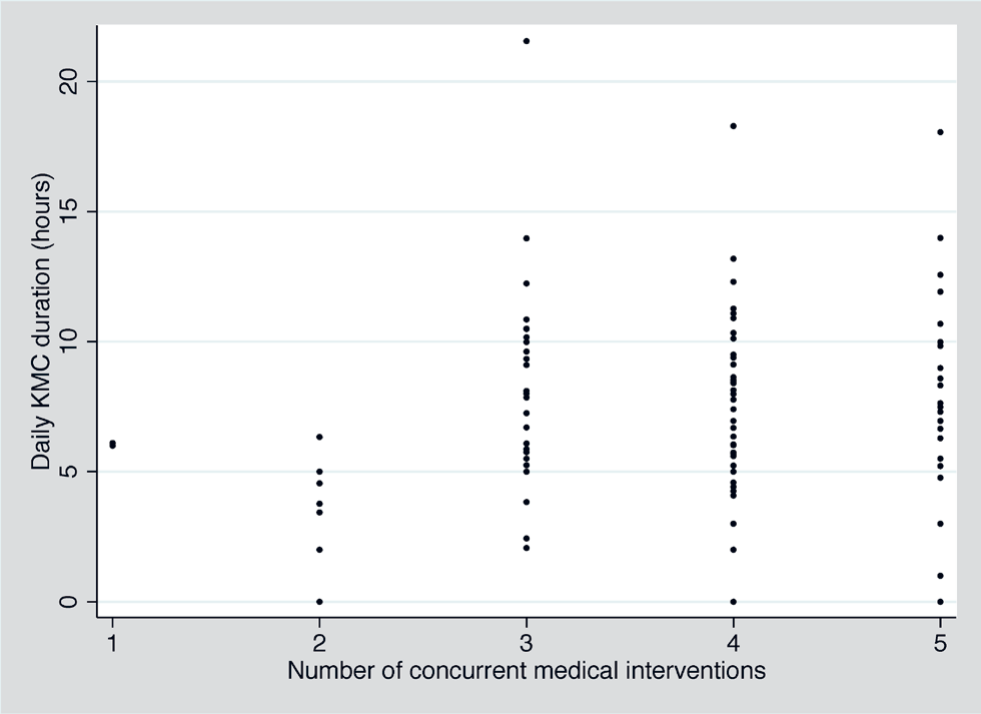
Daily kangaroo mother care (KMC) duration by number of concurrent medical interventions (N = 10).

Amongst the 10 participants, we observed 315 KMC episodes over 102 person-days. The median age at KMC initiation was 30.3 hours (IQR: 19.6-95.2 hours). The provider was the mother in 298 (94.6%), the father in 2 (0.6%), and another family member in 15 (4.8%). Amongst the 8 participants that breastfed during the first 14 days of admission, the median age at breastfeeding initiation was 4.5 days (IQR: 2.0-7.5 days). The mean number of concurrent medical interventions was relatively constant across the first 14 days of admission, ranging from 3.7 to 4.1 interventions per day (**Table 3**). The median duration of individual KMC episodes was stable across this time period, ranging from 115 to 134 minutes. The median daily duration of KMC ranged from 4.5 hours (day 3) to 9.7 hours (day 13) with a slight upward trend over time (**Figure 2**). Two neonates received the target duration of 18 hours on day 1 (18.1 and 21.6 hours) and one neonate received the target duration on day 5 (18.3 hours). The number of concurrent medical interventions an infant was receiving did not affect the daily duration of KMC (**Figure 3**).

**Table 3:** Concurrent interventions and KMC duration by study day (N = 10)

Study day	Mean (SD) number of interventions	Range number of interventions	Mean (range) number of KMC episodes	Median (IQR) duration of KMC episodes (minutes)	Median (IQR) daily duration of KMC (hours)
1 (n = 10)	4.0 (0.67)	3-5	2.4 (0-5)	120 (108-146.5)	5.1 (3.0-8.6)
2 (n = 10)	3.9 (0.88)	2-5	2.6 (0-5)	115 (65-140)	5.1 (0-8.4)
3 (n = 10)	4.1 (0.99)	2-5	2.0 (0-5)	120 (65-168)	4.5 (1.0-7.0)
4 (n = 9)	4.0 (1.0)	2-5	3.3 (0-4)	131 (120-196)	8.5 (6.7-9.5)
5 (n = 9)	4.0 (1.0)	2-5	3.3 (2-5)	120 (60-148)	7.4 (5.0-8.3)
6 (n = 9)	3.9 (1.05)	2-5	3.0 (0-5)	130 (100-156)	6.1 (5.0-7.3)
7 (n = 9)	3.7 (1.22)	1-5	3.4 (0-6)	120 (108-202)	8.1 (6.0-11.9)
8 (n = 9)	3.7 (1.41)	1-5	3.0 (0-4)	120 (108-146.5)	6.7 (4.4-10.3)
9 (n = 8)	3.5 (1.41)	1-5	3.1 (1-6)	120 (88-138)	6.3 (2.4-9.6)
10 (n = 4)	4.0 (0.82)	3-5	4.0 (2-5)	120 (75-146)	8.3 (6.0-9.8)
11 (n = 4)	4.0 (0.82)	3-5	4.0 (3-5)	129 (92-162.5)	9.3 (7.2-10.1)
12 (n = 4)	3.8 (0.96)	3-5	4.0 (3-5)	134 (80-155)	7.5 (6.5-10.8)
13 (n = 4)	3.8 (0.96)	3-5	4.5 (3-6)	132 (90-160)	9.7 (8.4-11.4)
14 (n = 4)	3.8 (0.96)	3-5	3.0 (2-4)	120 (84-163)	5.6 (4.1-8.4)

KMC – kangaroo mother care, SD – standard deviation, IQR – interquartile range

### 3) Acceptability sub-study

We enrolled 20 key stakeholders (10 parents, 10 providers). **Table 4** shows the participant characteristics.

**Table 4:** Acceptability study participant characteristics (N = 20)

Parent characteristics, n = 10		Provider characteristics, n = 10	
Age (median, range)	27 (22-35)	Age (median, range)	48.5 (29-55)
**Demographic characteristics**		**Role in Newborn Unit**	
Mother	8 (80%)	Paediatrician	2 (20%)
Married	7 (88%)	Charge nurse/nurse officer	2 (20%)
Resides in rural area	5 (63%)	Midwife	6 (60%)
Father	2 (20%)	Educational level	
Married	2 (100%)	Diploma	6 (60%)
Resides in urban area	2 (100%)	Certificate	2 (20%)
Educational level		Master’s degree	2 (20%)
Primary	3 (30%)		
Secondary	5 (50%)		
College	2 (20%)		

We use the main themes emerging from the data to structure the presentation of material from the interviews, with themes broadly classified as facilitators or barriers, as below.

#### Facilitators

##### General knowledge about KMC and its benefits

All parents and providers knew KMC is a method to provide warmth to preterm babies through SSC, usually with the mother.

*“It's a traditional or African way of keeping a baby warm; it is usually for babies that are born preterm. You put the baby on the chest of the mothers. It creates love between the mum and child.”* (Father, age 28)

While most parents reported that the mother is the KMC provider, several health care providers stated that other family members could also deliver KMC. Parents and providers agreed that KMC promotes breastfeeding and infant bonding. One mother also said KMC promotes connection between parents.

*“As a KMC father myself, I felt that there was a lot of bonding between myself and my daughter. She is 9 years old now. I feel like I have bonded with her more than my other children. Each time I meet someone, I proudly tell them, 'This is my daughter.' When people say, ‘so what,’ I tell them that she was 900 grams and look at how she has grown.”* (Paediatrician, age 52)

Half of providers mentioned that KMC promotes immunity and overall health in unstable preterm and LBW neonates. One provider described how they encourage mothers to practice KMC to help stabilise neonates when oxygen is unavailable.

*“When the power has gone, we encourage mothers to do it to help stabilize the breathing when the oxygen is off.”* (Midwife, age 29)

##### Improved monitoring of unstable neonates

The majority of providers felt that KMC leads to improved monitoring of unstable neonates, compared to incubator care.

*“While the baby is on KMC, the monitoring is better because the mum is always there. However, if the baby is an incubator, the nurse may not be able to check on the baby frequently because of the limited human resources available.”* (Paediatrician, age 55)

The majority of parents also felt that KMC leads to improved infant monitoring. Three mothers and one father additionally described how practicing KMC gave them a sense of responsibility in caring for their babies.

*“Because I want my baby to be looked after properly, I start to become responsible for my child and I find myself looking to see what's wrong and letting the doctor know.”* (Mother, age 27)

Almost half of providers stated that improved availability of monitoring devices would help facilitate KMC provision amongst unstable neonates.

*“If it's there, it would work. Something preferably with lights that's easier for mums to remember.”* (Charge nurse, age 50)

##### Provision of medical interventions in the KMC position

Seventy-five percent of parents and providers felt that medical therapies (eg, oxygen, IV fluids) can be provided to neonates while in the KMC position.

*“I don't think there are any concerns because the baby can face either side of the mother's chest while they are receiving oxygen and IV fluids.”* (Midwife, age 51)

##### KMC initiation

The majority of parents and providers felt KMC could be used in the first 48 hours after birth.

*“I think it's a good idea because that skin-to-skin contact will stabilise the baby's temperature faster than an incubator.”* (Midwife, age 51)

##### Staff and peer counselling

All providers and the majority of parents agreed that staff counselling is essential to promote KMC amongst unstable neonates. Three providers and three parents stated that peer counselling is a valuable approach to promote KMC in the hospital. One provider suggested a follow-up club where mothers could talk to each other about KMC and preterm care.

*“Peer counselling is key because another mum in the ward can share her experience; we have found that peer counselling is more important and effective than counselling from a doctor or nurse.”* (Paediatrician, age 55)

*“We like the follow-up clinic on Friday because it gives the mums a chance to talk amongst each other. You will often hear them ask “I put my child like this, is this the right way?” They also compare and contrast. We also encourage mothers to teach each other on the proper way to do it.”* (Paediatrician, age 52)

##### Family support and meal provision

Almost half of providers discussed the importance of family support, with two specifically commenting on the role of the mother in law in influencing KMC practice in facilities.

*“The management of the baby is a combination of the mum, the nurses and the doctors, but also the relatives. The mothers need support from the relatives to survive in the hospital.”* (Charge nurse, age 50)

One provider and one mother mentioned that provision of meals in the newborn unit would be helpful to mothers who lack family support and money.

##### Concerns related to incubators

Half of parents expressed concerns that incubators were unsafe, some stating they cause brain damage.

*“Sometimes I think an incubator can harm the baby's brain. There is no love, just heat, in the incubator.”* (Mother, age 22)

One provider and one mother mentioned the shortage of incubators in the newborn unit.

*“The incubators are not enough because sometimes your baby could be there and then the doctor comes in with a much sicker baby and they remove yours and the cycle continues like that until your baby never goes back to the incubator.”* (Mother, age 32)

##### Cost savings and sustainability of KMC

Three providers stated that KMC is less costly than incubator care. One mother mentioned the sustainability of KMC and one provider discussed the applicability of KMC across different income and education levels.

*“It is appropriate in every household regardless of someone’s level of civilization. We believe it has bigger advantages which is less cost and sophistication and less risks in terms of temperature and it is sustainable.”* (Paediatrician, age 52)

##### Community outreach and education

Three providers and two parents felt that provision of KMC education in antenatal clinics and elsewhere in local communities would help promote KMC practice amongst hospitalised neonates. One provider suggested that such outreach could involve mothers of former preterm neonates teaching members of their local community about KMC.

*“Some mums don't have sheets and hats to do KMC… they come unprepared. We provide the hats, but the sheets we don't have anymore. Some counselling and training on KMC needs to be done in the antenatal ward to prepare mums.”* (Nursing officer, age 50)

*“I believe KMC training should start as far as the rural areas; there are some health workers there I'm sure don't know about it. It would be beneficial; this training would then extend to health centre-3s* [lowest level facility in Uganda at which deliveries are allowable] *and go to antenatal care as well.”* (Father, age 28)

#### Barriers

##### Stigma and guilt related to prematurity

Several providers mentioned that stigma and guilt about having a preterm infant are common in the local communities.

*“The mothers that are getting preterm babies for the first time are reluctant to help because they don't believe that these babies can survive.”* (Charge Nurse, age 50)

*“Acceptability of this baby is an issue; some mums accept their babies while others, especially those that have had normal births in the past, are torn up about it.”* (Paediatrician, age 55)

Several mothers described personal observations about the high risk of death in preterm babies.

*“I have seen some babies die, especially those that range between 5 and 5.5 months. These babies are at a higher risk of dying than those that are 6 or even 7 months. They usually pass away.”* (Mother, age 32)

##### Concerns related to infant monitoring

Providers agreed that monitoring unstable neonates in the KMC position can be challenging due to staff shortages and/or lack of monitoring devices.

*“I worry sometimes because these mothers may not observe the baby; in some situations, the baby may fall inside without the mother noticing… we have to pay extra attention to these mums in the event they aren't monitoring their baby.”* (Midwife, age 51)

One mother expressed concern that her baby might be monitored less closely, but agreed that KMC allows mothers to learn more about the care of preterm neonates.

*“I'm very worried that doing KMC would not allow my baby to be monitored as much because the baby can change colour at any time and you aren't sure what's wrong. Sometimes as mothers, we don't know the difference in colour change. However, it also allows the mother to learn more about her baby and update the doctor or nurse.”* (Mother, age 32)

##### Concerns related to pain and dislodgement of tubing

A few mothers expressed concern that doing KMC while receiving oxygen or IV fluids could be painful for the baby or could lead to accidental dislodgement of tubing.

*“I want to be close to him and I feel like those tubes would hurt him as we are doing KMC.”* (Mother, age 26)

One mother discussed the difficulties of KMC provision following a caesarean delivery.

*“I had a caesarean section and I couldn’t move properly or even hold a baby for at least 3 or even 4 days so I wasn’t able to do KMC immediately.”* (Mother, age 30)

##### Lack of family support, finances, and time away from work

One mother stated that lack of family support and lack of money for mothers to buy food are common in the newborn unit. A father discussed difficulties with KMC related to cost and increased time away from work.

*“I was supposed to work but I had to get the day off and come here so that they could be discharged from the hospital because it's becoming very expensive. The more time we are here, the more money I have to spend.”* (Father, age 25)

##### Lack of beds, space, and privacy

All providers and 80% of parents felt that lack of beds and space in the newborn unit was a barrier to KMC practice. Twenty percent of providers and 80% of parents, including both fathers, perceived lack of privacy to be an issue.

*“You are a man and they are making you take off your shirt; I know men are shy. If there was a place with less people and you are free, that would be good.”* (Father, age 25)

##### Lack of KMC education

Half of parents felt that lack of education was a barrier to KMC provision in facilities.

*“Sometimes there are students here and they don’t tell us about KMC and then by the time we are learning it has been three days after!”* (Mother, age 23)

##### Lack of motivation amongst parents

Three providers described how it can be difficult to motivate mothers to devote sufficient time to KMC.

*“Some of these mothers don't find it realistic to sit with their babies for that period of time and ultimately don't enjoy it. They want it to be quick.”* (Midwife, age 29)

## DISCUSSION

To our knowledge, this is the first study to evaluate the incidence of clinical instability, as well as the feasibility and acceptability of KMC in this vulnerable population.

### Instability criteria

We found that the majority of neonates weighing ≤2000g admitted within 48 hours of birth in this regional hospital met our criteria for clinical instability. Based on a review of currently available evidence, we defined instability by the need for ≥2 medical therapies. For public health impact, it was crucial to select criteria that would permit inclusion of the majority of vulnerable neonates, who face the highest risk of mortality, while still ensuring safety. Importantly, these therapy-based criteria identified 89% of all LBW neonates and 90% of VLBW and ELBW neonates as unstable within 48 hours of birth. We hypothesised that these criteria would identify all ELBW neonates as unstable; however, it is possible that some administered therapies were not properly recorded in medical charts. There remains a need for an evidence-based approach to consistently define stability criteria in order to improve clinical care and facilitate research in this population [12,48].

### Feasibility

We found that it is feasible to monitor and provide medical interventions to unstable neonates in the KMC position. Underlining the vulnerability of these preterm neonates, two of the 10 enrolled neonates died during the 14-day enrolment period. Both were extremely premature and extremely low birthweight, thus at very high risk of death, particularly in a low-resource setting given the lack of ventilatory support and other intensive care usually required for survival at this gestational age. The median age at enrolment was 25.3 hours, and neonates received a median duration of KMC ranging from 4.5 to 9.7 hours per day with a slight upward trend over time. This is comparable to findings from several RCTs, which reported median daily durations of ≥4 to 10 hours with mean/median age at enrolment ranging from <1 to 4.7 days [15,21-24,28]. Notably, despite nurse counselling, few neonates achieved the target KMC duration of 18 hours per day (two on day 1 and one on day 3). Three RCTs evaluating continuous KMC reported durations of ≥20 hours per day with mean/median age at enrolment of 4 days [17], 10 days [16], and 13 days [30]. Studies have shown that continuous KMC can be difficult to achieve as mothers may find it overwhelming, and clinicians and administrators may be unaware of the need for near-continuous provision [49]. In this study, the number of concurrent medical therapies an infant was receiving did not affect the daily duration of KMC. The median duration of individual KMC episodes was approximately 2 hours across the study period. Experts have suggested that a minimum episode duration of 2 hours is important [9] because it provides the stimulation needed to increase milk volume and facilitate let-down; facilitates the infant spontaneously awakening, self-regulating feeding, and completing a sleep cycle [50,51]; and reduces the number of transfers into and out of the KMC position [52].

### Acceptability

We found that stigma and guilt related to having a preterm baby is common in local communities in southeastern Uganda. Another study in this region found that most of the mothers interviewed believed that preterm babies could survive if treated properly. However, that study also found that some mothers wished their preterm neonates had never been born [53]. Other studies have also reported stigma, fear, shame, or guilt in association with having a preterm infant [54–56]. All participants knew that KMC provides warmth to small or preterm neonates. Participants were also aware that KMC promotes breastfeeding, stimulates breathing, and promotes infant bonding. Other studies in low-resource settings have also reported knowledge of these benefits [56-58]. In particular, the effect of KMC on parent-infant bonding has been widely reported [9,59-63]. In this study, most parents stated that the mother is the KMC provider. In another Ugandan study, most men interviewed felt that women were the sole KMC providers [53]. The majority of parents and providers found use of KMC among neonates who are receiving concurrent medical therapies in the first 48 hours after birth acceptable. A few mothers expressed concern that KMC might cause pain or displace IV/oxygen tubing, worries that have also been seen in neonatal units in high- and upper middle-income settings [64-67]. The majority of parents and providers felt that KMC leads to improved infant monitoring. Other studies have also found that parents feel more responsible for the health of their neonates when providing KMC [9,56].

Our study found that lack of beds/space, privacy issues, insufficient staff and devices for monitoring, inadequate KMC education, and difficulties motivating mothers to devote time were the most common barriers to KMC practice. Other studies have also reported that lack of space, privacy, and KMC resources hindered KMC practice [54,68-72]. A study in Malawi found that lack of recreational activities was an obstacle [57]. Parents and providers suggested that KMC practice could be improved through staff and peer counselling, more beds/space, improved availability of monitoring devices, family support, and community outreach. Several studies have noted the importance of staff training and counselling on KMC [73,74], and a related RCT demonstrated the efficacy of peer counselling in promoting breastfeeding amongst admitted preterm neonates [75]. Other studies have noted the importance of family support, particularly from the father and mother in law [49,57,63,76], and other social support, such as from peers and nurses, in promoting KMC [49,77,78]. In support of the recommendation for community outreach, a study found that zero of 16 health centres in two districts of eastern Uganda promoted KMC practice. Further, local community members had minimal knowledge about KMC [53].

### Limitations

This study has limitations. Audit findings are limited especially by incomplete records, a common issue in low-resource contexts. To address the latter, we excluded charts that were not satisfactorily complete, which were defined as including both birthweight and medical therapies administered. Our feasibility findings are based on a sample of only 10 neonates, which is too small to draw firm conclusions. Importantly, however, we continuously observed clinical care and KMC practice from the time of enrolment to discharge, up to a maximum of 14 days. In a recent systematic review, only 12% of included studies reported the duration of KMC [79]. For the acceptability sub-study, we interviewed 10 parents (8 mothers and 2 fathers) and 10 providers who had experienced a preterm birth or cared for preterm neonates, respectively. Participants were assured that their responses were confidential in nature, and would not affect the care of their infant in any way; however, social desirability bias may have influenced the responses of some parents.

## CONCLUSIONS

This study demonstrates that KMC for neonates meeting criteria for clinical instability, defined as the need for ≥2 medical therapies in the first 48 hours, was feasible in a small sample of neonates weighing ≤2000g and acceptable to parents and providers at Jinja Hospital in Uganda. To improve clinical care and facilitate research, there remains a need for an evidence-based approach to consistently define stability criteria for KMC. Further, RCTs are crucial to examine the effect of KMC on survival in this vulnerable population. Such evidence would have broad applicability, especially in low-resource settings where most neonatal deaths occur.
